# Antioxidant Properties of Lapachol and Its Derivatives and Their Ability to Chelate Iron (II) Cation: DFT and QTAIM Studies

**DOI:** 10.1155/2020/2103239

**Published:** 2020-03-31

**Authors:** Djafarou Ngouh Pajoudoro, Daniel Lissouck, Baruch Ateba Amana, Joseph Zobo Mfomo, A. E. B. Abdallah, Alfred Aristide Flavien Toze, Désiré Bikele Mama

**Affiliations:** ^1^Department of Chemistry, Faculty of Science, University of Douala, P. O. Box 24157, Douala, Cameroon; ^2^Department of Physics, Faculty of Science, University of Douala, P. O. Box 24157, Douala, Cameroon; ^3^Department of Forestry and Wood Engineering, Advances Teachers Training College for Technical Education, University of Douala, P. O. Box 24157, Douala, Cameroon

## Abstract

The elucidation of the complexation of lapachol and its derivatives to Fe^2+^ cation has been done using the density functional theory (DFT). This complexation has been limited to bidentate and tridentate to Fe^2+^ cation. Geometry optimizations have been implemented in gas and solution phase (water, acetonitrile, chlorobenzene, benzene, and toluene) for ligands at B3LYP/6-311++G (d,p) level of theory using B3LYP/6-31+G(d,p) optimized data as starting point. But, the geometrical optimizations in solution phase of the 22 complexes analyzed of lapachol and its derivatives to Fe^2+^ cation were restricted to acetonitrile and benzene. The complexation energy and the metal ion affinity (MIA) have also been calculated using the B3LYP method. The results obtained indicated a proportionality between the MIA values and the retained charge on Fe^2+^ cation for *k*^2^-(O_1_,O_2_) modes. But, an inverse proportionality has been yielded between these two parameters for *k*^3^-(O_2_, C=C) tridentate modes. For k^3^-(O_3_,C=C) tridentate mode coordination, the higher stability has been obtained. In this latter tridentate coordination in gas phase, the topological analysis of complexes exhibits the fact that the electron density is concentrated between the O_3_ oxygen atom of the ligand attached to Fe^2+^ and this metal cation. Moreover, the hydrogen bond strength calculated for isolated ligands (situated between 23.92 and 30.15 kJ/mol) is in the range of normal HBs. Collectively, all the complexation processes have shown to be highly exothermic. Our results have also shown that the electron extraction from **Fe**^**2+**^**...La**_**i**_ complexes is more difficult compared to that from free ligands.

## 1. Introduction

Lapachol (2-hydroxy-3-(3-methylbut-2-enyl) naphthalene-1,4 dione is a natural quinone isolated from Bignoniaceae family found in Brazil [1] (more specifically from bark extracts of *Tabebuia sarratifolia* [2]). The interest of our attention resulted from its extensive range of biological and pharmaceutic activities [3–5]: antitumor, antibiotic, antimalaria, trypanocidal, and leishmanicidal activities. Its ability to interfere with the bioactivities of topoisomerases (set of enzymes critical for DNA replication in the cell) justifies the intense investigation of its derivatives [6]. Moreover, its higher activity against carcinosarcoma tumor (Walker 256) [7] has prompted Hartwell and Abbot to examine the efficiency of its derivatives. In the same vein, studies on the activity against carcinosarcoma (Walker 256) and that on the structural modifications of these derivatives have been reported experimentally and theoretically [8]. Silva et al. [9] suggested that this evident cytotoxic property is due to the aromaticity of naphthalenic ring of the molecular systems examined. The antioxidant ability of lapachol has been experimentally confirmed by Sadananda et al. through the isolation and characterization of lapachol from different parts of *Tabebuia* a*rgentea* [10]. The excellent chelating keto-enol function of lapachol and its derivatives has been proved [11, 12]. In addition, the enhancement of antiplasmodial activity has been observed by the ruthenium chelation of free lapachol by Barbosa et al. [13]. Other modifications of the activity of lapachol resulting from the metal chelation have been experimentally tested using the manganese (II) lapacholate polymer. In the same vein, Tabrizi et al. have synthesized and characterized 3^rd^ row metal (Cu, Co, and Ni) complexes of lapachol that have exhibited a promising antitumor activity [14]. These three complexes were previously characterized by cyclic voltammetry [15]. In addition, the antimony (V) and bismuth (V) complexes of lapachol have shown a significant cytotoxic activity [16]. Despite this fact, a deep analysis of the influence of the metal chelation on the biological activities of free lapachol is lacking. Such an examination (of the antioxidant activity for instance) may give plausible explanations of the effectiveness of the intake of lapachol tea for skin antiaging effect [17]. To the best of our knowledge, few computational studies [8] concerning the antioxidant activity of lapachol and its derivatives have been reported. Until now, there is no study on the impact of the metal chelation of these molecular systems on their antioxidant powers.

The characterization of the antioxidant activity for large number of antioxidants **RX-H**, [18–22] has been done by the calculation of the **RX-H** (X = donor atom such as O, N, ...) bond dissociation enthalpy (BDE). From these calculations, the prediction of the relationship structure-activity and the design of novel possible antioxidant have been carried out. The radical formed from the hydrogen atom transfer (HAT) can be obtained through two-step mechanisms: the single electron transfer-proton transfer (SET-PT) mechanism (expressed using ionization potential (IP) and proton dissociation enthalpy (PDE) of the RX-H^•+^ radical cation) and the sequential proton loss-electron transfer (SPLET) mechanism that matches up with the proton affinity (PA) of the anion RX^−^ and the reaction enthalpy of abstraction of an electron from this anion denoted as ETE (electron transfer enthalpy). The ability of the potential antioxidant to chelate transition metal ions, precisely iron and copper cations (in order to prevent the damage of oxidative stress from the participation in free radicals generation [23]), is also considered as antioxidant mechanism. The metal chelation reduces the production of toxic hydroxyl radical (OH•) from Fenton reaction:

(1) $$\begin{array}{c} H_{2}O_{2}+M^{n+}®M^{\left(n+1\right)+}+HO^{-}+HO^{\times } \end{array}$$

**Figure 1:**
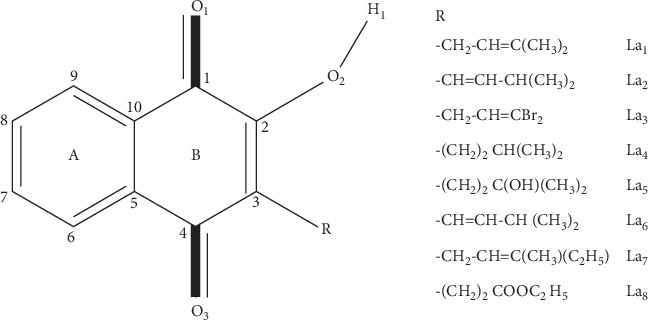
Numbering system used for **La**_i_ free ligand in their various coordination modes.

The current study reports the results of a computational examination of the antioxidant power of molecular system in different media (gas and solution phase) and the influence of the iron (II) chelation. A systematic study of reaction enthalpies related to SET mechanism for lapachol and its seven derivatives (Figure 1) denoted as **La**_**n**_ (*n* = 1–8) is performed. A direct verification of accuracy of calculated reaction enthalpies cannot be performed due to the nonavailability of their experimental homologues. The calculated physiochemical antioxidant descriptors (**IP**) for this molecular system are compared to that of the classical antioxidants (gallic acid, caffeic acid, ferulic acid, ascorbic acid (vitamin C), and trolox). The metal cation affinity for Fe^2+^ cation and the impact of this metal chelation on the antioxidant activities through the examination of the SET mechanism are also inserted. This last point must be helpful for the clarification of the properties of **La**_**n**_**−Fe**^**2+**^ that may facilitate the selection of appropriate chelating and scavenging agent for the chelating therapy of Alzheimer [24].

## 2. Computational Details

The geometry optimizations of lapachol and its seven derivatives molecular systems were performed at the B3LYP/6-311++G(d,p) level using Gaussian 09 [25] program from B3LYP/6-31+G(d,p) data considered as starting point. Collectively, these two series of geometry optimizations were carried out with no symmetry constraints in the ground state. The frequency calculations have been done to select optimized structures without negative frequencies. Free ligands (RX-H) were optimized in gas phase and in five solvents (water, acetonitrile, chlorobenzene, benzene, and toluene). For these latter cases, the integral equation formalism of polarizable continuum model **(**IEF-PCM**)** [26, 27] was used. The ionization potential (IP) of these free ligands (enthalpy change of the reaction (2)) is considered as descriptor of the SET **(**single electron transfer) antioxidant mechanism:

(2) $$\begin{array}{c} RX-H®RXH^{-}+e^{-}\left(IP\right) \end{array}$$

Higher antioxidant activity of the molecular library is the molecule that exhibits lower (IP) values. This molecular descriptor was therefore calculated using the following formula

(3) $$\begin{array}{c} IP=H\left(RXH^{.+}\right)+H\left(e^{-}\right)-H\left(RXH\right) \end{array}$$

The gas phase enthalpy for electron (**e**^**−**^) is equal to 3.145 kJ·mol^−1^ [28]. We have adopted the solution phase enthalpies proposed by Markovic et al. [29]. The ionization potential free energy (IPFE) which is the reaction free energies of reaction (2) is calculated according to the following formula:

(4) $$\begin{array}{c} IPFE=G\left(RXH^{.+}\right)+G\left(e^{-}\right)-G\left(RXH\right) \end{array}$$

where the calculated gas free energy of an electron **G**(**e**^**−**^) is equal to −3.72 kJ/mol [30]. Electron solvation free energies were taken from the literature [29].

From the enthalpy change of the complexation (5) of lapachol and its seven derivatives by **Fe**^**2+**^, the **La**_**i**_**−Fe**^**2+**^ binding energy (*i* = 1–8) denoted by **E**_**int**_ can be predicted:

(5) $$\begin{array}{c} La_{i}+Fe^{2+}⟶La_{i}\ldots Fe^{2+} \end{array}$$

(6) $$\begin{array}{c} E_{int}=E_{La_{i}\ldots Fe^{2+}}-E_{La_{i}}-E_{Fe^{2+}} \end{array}$$

where **E**_**La**_**i**_…**Fe**^2+^_, **E**_**La**_**i**__, and **E**_**Fe**^2+^_ represent, respectively, total energy of the optimized complex (**E**_**La**_**i**_…**Fe**^2+^_), isolated ligand (**La**_**i**_), and free iron (II) cation. The enthalpy (ΔH) and free enthalpy ((ΔG) change were also predicted. The ionization potential of these complexes (IP_C_) as enthalpy change of the reaction (7) was calculated according to (8). This fact has favored the assessment of such a complexation on the antioxidant mechanism in various media through its influence on SET mechanism:

(7) $$\begin{array}{c} La_{i}\ldots Fe^{2+}⟶La_{i}\ldots Fe^{3+}+e^{-} \end{array}$$

(8) $$\begin{array}{c} IP_{C}=H\left(La_{i}\ldots Fe^{3+}\right)+H\left(e^{-}\right)-H\left(La_{i}\ldots Fe^{2+}\right) \end{array}$$

where **H**(**La**_**i**_**...Fe**^**n+**^) is the formation enthalpy of the complex **La**_**i**_**...Fe**^**n+**^ (*n* = 2 and 3).

In general, the ionization values can be directly obtained as the negative of the eigenvalue of highest occupied molecular orbital (**HOMO**) (**IP** for **La**_**i**_ ligands and **IP**_**C**_ for **La**_**i**_**…Fe**^**2+**^ complexes) as recommended by Koopman's theorem [31]. But, this procedure has been adopted exceptionally for **La**_**i**_**…Fe**^**2+**^ complexes (**IP**_**C**_) due to the difficult estimation of the geometry changes provoked by the reflections or the relaxations of ligand fragment during the geometrical optimization progression.

The quantum mechanics atom in molecule (QMAIM) theory was consequently performed from the B3LYP/6-311++G(d,p) optimized structures to investigate these two parameters (bonding properties and bond critical points). More specifically, the clarification of the strength of hydrogen bond interactions (in isolated ligands) and metal-ligand interactions in complexes has been done using the Multiwfn program [32]. For bonding interactions, the indicators are electron densities BDE=*H*(*TrX*^•^)+*H* and its Laplacian ∇^2^*ρ*(*r*) at bond critical points (CP). The total number of these CPs obtained is in accordance with the Poincare–Hopf rule [33]. For deeper examination, the density of the total energy of electrons (H) defined as the sum of the Lagrangian kinetic electron density (G) and the potential electron density (V) at bond critical points (BCPs) has been estimated:

(9) $$\begin{array}{c} H_{BCP}=G_{BCP}+V_{BCP}, \end{array}$$

where **G**_**BCP**_ and **V**_**BCP**_ are, respectively, defined in (10) and (11):

(10) $$\begin{array}{c} G_{BCP}=\frac{3}{10}3{\left(\pi \right)}^{2/3}\rho {\left(r\right)}^{5/3}+\frac{1}{6}\rho \left(r\right), \end{array}$$

(11) $$\begin{array}{c} V_{BCP=\frac{\hbar }{4m}\nabla^{2}\rho \left(r\right)-2G_{BCP}}. \end{array}$$

The interatomic interaction energy (denoted as **E**_**in**_) in isolated ligands and in complexes was predicted by Espinosa approach [34]:

(12) $$\begin{array}{c} E_{int}=\frac{1}{2}V_{BCP}. \end{array}$$

## 3. Results and Discussion

### 3.1. General Considerations on Isolated Ligands

The optimized structure for lapachol (**La**_**1**_) is characterized by planarity of the naphthalenic ring. This plane also contained the carbon atom through which the 3-methylbut-2-enyl substituent is connected to this ring. But the whole molecular system is not planar due to the free rotation about 113.7° around the C-C single bond bound to the C=C double bond. The substitution of the two methyl groups by the bromide atom on the lateral chain (**La**_**3**_) provokes an augmentation of this rotation angle about 11°. A migration of the C=C double bond toward the naphthalenic ring of the lapachol leads to an optimized structure (**La**_**2**_) characterized by the C=C double bond contained in the plane formed by this ring. Such rigidity induces the symmetrical position of the two methyl groups according to the preceding plan. The substitution of the unsaturated substituent by the 3-methylbutyl (**La**_**4**_) or 3-hydroxy-3-methylbutyl substituent has yielded optimized structures with a lateral chain almost perpendicular to naphthalenic ring. On the whole, optimized structures obtained in gas phase are characterized by the formation of hydrogen bond (HB) between the hydrogen atom (H_1_) of hydroxyl group (H_1_-O_2_) and the oxygen atom of the carbonyl group (C_1_=O_1_). This formation of hydrogen bonds is in agreement with the cutoff definition of X-H...A hydrogen bonds proposed by Steiner [35] (H...A > 3.0 Å and X-H...A angle > 110°: [Supplementary-material supplementary-material-1]).

### 3.2. Metal Chelation Mechanism

**Figure 2:**
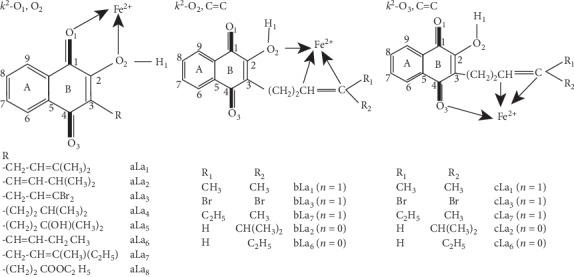
Numbering system used for Fe^2+^...La_i_ complexes in their various coordination modes.

Our results reveal the fact that Fe^2+^ cation is bound to **La**_**1-8**_ ligand through bidentate or tridentate connection. The bidentate attachment (denoted **k**^**2**^**-O**_**1**_**,O**_**2**_) is observed when Fe^2+^ cation is placed in vicinity of sp^2^ oxygen (O_1_) and sp^3^ oxygen (O_2_) of the O_2_-H_1_ group (Figure 2). The optimized structures of **Fe**^**2+**^**...La**_**i**_ complexes obtained denoted as **aLa**_**i**_ (*i* = 1–8) exhibited a Fe^2+^ cation contained in the plane formed by the naphthalenic ring. This structure is characterized by Fe-O_1_ bond distances longer than Fe-O_2_ ones (average difference is 0.087 Å). Collectively, the optimization of **k**^**2**^**-O**_**1**_**, O**_**2**_ complexes leads to structures where the hydrogen atom (H_1_) shifts to be 10.2–29.4° off-plane. We have also integrated two possibilities to bind Fe^2+^ cation for **La**_**5**_ ligand: (i) to bind to Fe^2+^ cation to sp^3^ O_2_ oxygen atom of the O_2_-H_1_ group and sp^3^ O oxygen atom of the O-H group of the R substituent of **La**_**5**_ ligand (**k**^**2**^**-O**_**3**_**,O: ala**_**52**_**)** and (ii) to bind to Fe^2+^ cation to sp^2^ O_3_ oxygen atom of the naphthalenic system and sp^3^ O oxygen atom of the O-H group of the R substituent of the ligand concerned (**k**^**2**^**-O**_**3**_**,O: ala**_**53**_**)**. In the former case, the breakage of **O**_**1**_**⋯H**_**1**_**-O**_**2**_ hydrogen bond is not observed, but the H_1_ proton is transferred to O_1_ and then becomes closer to O_1_ atom than to O_2_ atom. The simultaneous binding of Fe^2+^ cation to sp^2^ O_2_ oxygen atom of the O_2_-H_1_ group and C=C π bond of the substituent R (**k**^**3**^**-O**_**2**_**,C=C**) (Figure 2) has also been observed. Similar tridentate attachment (**k**^**3**^**-O**_**3**_**,C=C**) of Fe^2+^ cation to sp^2^ O_3_ oxygen of the naphthalenic moiety and C=C π bond of the same **La**_**1-8**_ ligand is possible. For sake of clarity, these complexes have been, respectively, symbolized by **bLa**_**i**_ and **cLa**_**i**_ (*i* = 1-8). From the input geometry of the **bLa**_**2**_ complex, the optimized structure is characterized by Fe^2+^ ion bound to **La**_**2**_ ligand through a bidentate attachment. In order to evaluate the importance of hydrogen bonds, we have built initial geometrical structures with hydrogen bond or not (in both cases). The introspection of optimized structures obtained reveals the fact that the variation of Fe^2+^-ligand bonds (0.010–0.014 Å) is minor. This fact showed that the insertion of the O_1_⋯H_1_-O_2_ hydrogen bond in the **kLa**_**i**_ (*k* = *b*,c) does not give any additional information on geometrical parameters. On the whole, Fe-O_i_ (*i* = 1, 2) bond lengths are longer than Fe-C homologues.

For **La**_**3**_ ligand, the lowest total energy corresponding to the **cLa**_**3**_ complex indicates the fact that **k**^**3**^**-(O**_**3**_**,C=C)** coordination mode yields the most stable complex. Similar observations have also been obtained for other ligands (**La**_**2**_**, La**_**6**_, **and La**_**7**_) in gas phase. This higher stability obtained for such a coordination mode is attributed to simultaneous attachment of Fe^2+^ cation to C=C and sp^2^ O_3_ oxygen atom. The latter exhibits greater electron donor nature compared to sp^3^ O_2_ oxygen atom. This fact is in accordance to Cu^2+^ binding to 2-methyl-1-(2,4,6-trihydroxy-3-(3-hydroxy-3,7-dimethyloct-6-enyl)phenyl)propan-1-one, called Hyperjovinol extract from *Hypericum jovis* [36]. The significant relative energy obtained for **k**^**2**^**-O**_**1**_**,O**_**2**_ confirmed the augmentation of the stability of complexes in multidentate chelation of Fe(II) previously enlightened by Mwadham et al. [37]. The higher stability of **aLa**_**53**_ compared to that of other **Fe⋯La**_**5**_ complexes (**aLa**_**5**_ (219,782 kJ/mol higher in total energy than **aLa**_**53**_) and **aLa**_**52**_ (28,209 kJ/mol higher in total energy than **aLa**_**53**_) also displayed the sensitive influence of the type of electron donor group on which the Fe^2+^ cation is connected. In order to have much improved understanding of the stability, the interaction energies, interaction enthalpies, and the Gibbs energies of complexation have been calculated at B3LYP/6-311++G(d,p) level. The obtained results in the two solvents adopted (acetonitrile and benzene) for complexation and that obtained for gas phase are shown in Table 1. Collectively, all the interaction energies are highly negative, demonstrating that the complexation process is highly exothermic. The sensitive drop of binding energies observed when passing from gas phase to solvent used is attributed to solvent effect that obstructs the interaction between Fe^2+^ cation and **La**_**i**_ ligands. This drop is more significant for acetonitrile. For a more measureable description of the ligands' affinity toward Fe^2+^ cation, Table 1 displays that the selectivity on the basis of the complexation free energy is very versatile when passing to one coordination to another: an increasing ligand affinity is ordered as **La**_**4**_ < **La**_**5**_ < **La**_**3**_ < **La**_**2**_ < **La**_**6**_ < **La**_**7**_ (for **k**^**2**^**-O**_**1**_**,O**_**2**_ mode), **La**_**7**_ < **La**_**2**_ < **La**_**1**_ < **La**_**3**_ < **La**_**6**_ (for **k**^**3**^**-O**_**2**_**, C=C** mode), and **La**_**2**_ < **La**_**3**_ < **La**_**1**_ < **La**_**6**_ < **La**_**7**_ (for **k**^**2**^**-O**_**3**_**, C=C** mode) in gas phase. The versatility observed is due to diverse geometry changes of **La**_**i**_ ligands within the geometrical structures of **Fe**^**2+**^**...La**_**i**_ complexes during the optimization geometry. On the whole, the negative values of complexation free energy support the finding that this process is spontaneous in gas phase and two solvents used (acetonitrile and benzene).

**Figure 3:**
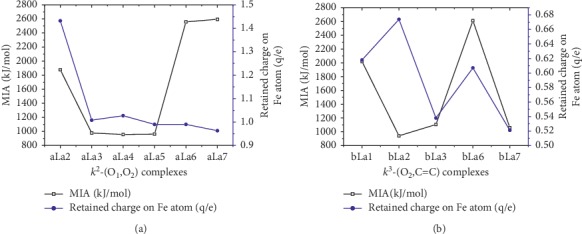
Correlation between the MIA (kJ/mol) and retained charge (Q/e) of Fe in **k**^**2**^**-(O**_**1**_**,O**_**2**_**)** and **k**^**3**^**-(O**_**2**_**,C=C)** coordination mode.

**Figure 4:**
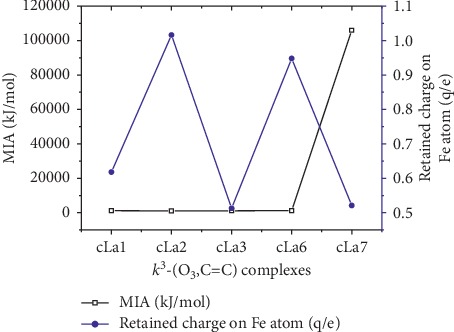
Correlation between the MIA (kJ/mol) and retained charge (Q/e) of Fe in **k**^**3**^**-(O**_**3**_**,C=C)** coordination mode.

Figure 3 indicates a possible correlation between the retained NBO charge on the Fe atom and the metal ion affinity (MIA) which is assumed to be the negative of the enthalpy change during the complexation process: in the case of **k**^**2**^**-(O**_**1**_**,O**_**2**_**)** mode, MIA values vary proportionately with the retained charge on Fe^2+^ cation. On the other hand, for **k**^**3**^**-(O**_**2**_**, C=C)** mode, MIA values vary inversely with the retained charge on Fe^2+^ cation. In the both cases, an exception is made for a**La**_**i**_ (*i* = 6 and 7) complexes.  Figure 4 exhibits the fact that there is no correlation between MIA values and retained charge on Fe^2+^ cation for k^3^-(O_3_, C=C) complexes. The values of the net charge of Fe^2+^ cation in the range + (1.432–0.513)e approve the fact that the Fe^2+^ cation experiences a reduction while the **La**_**i**_ ligands undergo an oxidation. The electron transfer from the latter to Fe^2+^ cation which augments with the attachment of Fe^2+^ cation to C=C confirms the oxidation role of the metal cation toward the molecular system examined. This result is in accordance with previous observations done on 1,2,3-triazol derivatives [38], curcumin [24], and Hyperjovinol [36]. From Table 1, one could notice that the diminution of the electron transfer is more sensitive in acetonitrile. This can be attributed to its polar property. On the whole, this is a suggestion that electron transfer from ligand to Fe^2+^ cation happens during the formation of coordination bond.

### 3.3. Topological Properties

The values of Laplacian of electron density (∇^2^*ρ*(*r*)) calculated (BCPs) for X-H...A bond critical points (BCPs) are a second derivative. The local electronic charge (depletion) is observed in the regions in which the values of Laplacian of electron density are positive ([Supplementary-material supplementary-material-1]). This reveals the fact that the interaction is characterized by the depletion of the density in the region of contact of hydrogen atom H_1_ and oxygen atom O_1._ The local excess in the kinetic energy is predominant in such an interaction. The average ratio −G_BCP_(r)/V_BCP_(r) greater than 1 in various media (gas (1.15), water (1.14), acetonitrile (1.18), benzene (1.19), and toluene (1.16)) confirms the noncovalent bonding interaction. The formation of hydrogen bonds is confirmed by positive values of small value of *ρ* and positive values of ∇^2^*ρ*(*r*) for H_BCP_ ([Supplementary-material supplementary-material-1]). Our data exhibit a linear correlation between these two parameters and the bond distance ([Supplementary-material supplementary-material-1]) in gas phase. The correlation coefficients (*R*^2^) were, respectively, equal to 0.98836 and 0.98096. This trend has been obtained in solution phase. In benzene for instance, these coefficients become, respectively, equal to 0.99951 and 0.99592. The inverse proportionality between hydrogen bond distances and these two topological parameters indicates an attenuation of orbital overlap that provokes an augmentation of the interatomic distances. As it is seen in [Supplementary-material supplementary-material-1], HB strengths calculated in the 23.92–30.15 kJ/mol range are in agreement with that of normal HBs (8.36–41.8 kJ/mol) [39].

The nature of interactions between the donor ligand and Fe^2+^ cation in the **Fe**^**2+**^**...La**_**i**_ complexes has been examined using the topological parameters listed in Table 2: the electron density (*ρ*(*r*)), its Laplacian ∇^2^*ρ*(*r*), kinetic energy density *G*(*r*), potential energy density *V*(*r*), total energy of electron (*H*(*r*), and ellipticity of the metal-ligand bond (*ɛ*)) at their bond critical points (BCP). The electron density of BCP for coordination bonds is found to be varied in the range: 0.069–0.111 Å′, 0.119–0.330 Å′ and 0.049–0.330 Å′, respectively, for **k**^**2**^**-(O**_**1**_**,O**_**2**_**)**, **k**^**2**^**-(O**_**i**_**,O)** and **k**^**3**^**-(O**_**i**_**, C=C)**: *i* = 2, 3. A survey of results of Table 2 demonstrates that the values of Laplacian of the electron density are collectively positive with exception made for **aLa**_**52**_ complex. This implies the preeminent interaction of the contraction of the electron density *ρ*(r) toward each nucleus. Therefore, the parallel gradient and the curvature of *ρ*(r) are great. The net forces of repulsion act on the nuclei. It is noteworthy to mention that *ρ*(r) values of Fe–O_i_ (*i* = 2, 3) bonds are higher than those Fe–C in the case of **k**^**3**^**-(O**_**2**_**, C=C)** and **k**^**3**^**-(O**_**3**_**,C=C)** coordination mode, supporting the fact that the bond distances of the latter are greatest.

The calculated average ratio −G_BCP_(r)/V_BCP_(r) is equal to 0.9670, 0.8108, and 0.8081 for **k**^**2**^**-(O**_**1**_**,O**_**2**_**)**, **k**^**3**^**-(O**_**2**_**, C=C),** and **k**^**3**^**-(O**_**3**_**,C=C)** coordination mode, respectively. These ratios comprised between 0.5 and 1 jointed to low values of *ρ*(*r*) and collective positive values of ∇^2^*ρ*(*r*) indicate the partially covalent nature of the metal-ligand bonding in gas phase. Similar results have been computationally obtained by Nkungli and Ghogomu through an investigation of the binding Fe^3+^ cation (protoporphyrin IX) to 4-methoxyacetophenone thiosemicarbazone) [40]. Additive investigations based on the values (|*λ*_1_|/*λ*_3_) at BCP (*λ*_1_,  *λ*_2_, and  *λ*_3_: eigenvalue of the Hessian matrix [40]) have been done to have further qualitative information on type of metal-ligand bonding interaction. A bond critical point is represented by one positive and two negative eigenvalues. For **k**^**2**^**-(O**_**2**_**,O)** coordination mode, the (|*λ*_1_|/*λ*_3_) values that range from 0.552 to 0.621 confirm the noncovalent nature of the Fe^2+^-ligand interaction. Contrary to **k**^**2**^**-(O**_**3**_**,C=C)** mode in which the (|*λ*_1_|/*λ*_3_) > 1 for Fe^2+^-O_3_ interaction (in almost complexes): 8,605 (**cLa**_**1**_), 6,341 (**cLa**_**3**_), 5,100 (**cLa**_**6**_) and 6,641 (**cLa**_**7**_).

Therefore, the concentration of electron density is located between the ligand atoms attached to Fe^2+^ and the latter. For all interaction analyzed, the negative values of *H*(*r*) obtained from our calculations exhibited the accumulation of electron density at the bond critical point. The highest value of ellipticity of the bond ɛ (*ε*=(*λ*_1_/*λ*_2_) − 1) is obtained for Fe-O_3_ bond in aLa_53_ complex ((*ɛ* = 1.893919).

### 3.4. Electron Transfer Mechanism

The calculated IP values obtained from equation (3) and Koopman's theorem for isolated ligand examined in various media are listed in [Supplementary-material supplementary-material-1]. The introspection of [Supplementary-material supplementary-material-1] indicates that IP values obtained for direct procedure (Koopman's theorem [31]) are lower in gas phase. The difference in IP ranges from 58.362 to 137.224 kJ/mol. Similar observations have been found out for nonpolar solvent (benzene and toluene) with lower differences in IP: an average difference in IP is 32.451 and 27.367 kJ/mol, respectively, for benzene and toluene. Nonetheless, higher average differences in IP (155.622 and 135,733 for water and acetonitrile respectively) have been yielded in the benefit of that obtained from Koopman's theorem. On the whole, we can conclude that the IP gap between results obtained from one procedure to another varies with the dielectric constant of the solvent. This may be attributed to the variation of the enthalpy of the solvation of the electron. Arbitrarily, we concentrate the further analysis of the antioxidant power of isolated ligand according to equation (3). From [Supplementary-material supplementary-material-1], it can be perceived that the shift of the C=C double bond toward the naphthalenic ring of **La**_**1**_ leads to a 9.84 kJ·mol^−1^ drop in IP (**La**_**2**_). This can be attributed to the extension of the conjugation system. This extension associated to conjugation of the π-electron has previously shown their influence on IP values of polyphenolic deoxybenzoins [41]. Recently, the effect of the double bond position on antioxidant power of 2-phenyl-benzofuran has been experimentally enlightened by Li et al. [42]. On the contrary, the substitution of the two methyl groups of **La**_**1**_ by bromide atoms provokes a 36.66 kJ/mol augmentation in IP (**La**_**3**_). In the same vein, the saturation of the C=C double bond of **La**_**1**_ results in a 46.70 kJ/mol increase in IP (**La**_**4**_). This augmentation illustrates the important role of this C=C double in improving antioxidant capacity. This is in agreement with enhancement of biological effect by the presence of C=C bond demonstrated by Chen [43]. The difference in IP for **La**_**2**_ and **La**_**6**_ equal to 4.776 kJ/mol exhibits the effect of steric hindrance on antioxidant activity. The alteration of the antioxidant activity of some Schiff base ligands and their copper (II) complexes by this steric hindrance has been reviewed by Salga et al. [44]. Similarly, Sun et al. have experimentally demonstrated that the antioxidant activity of flavonoids isolated from *Hebei balmy chrysanthemum* is sensitive to the steric hindrance of glucoside [45]. From [Supplementary-material supplementary-material-1], it should be observed that molecules with lower IP value than that of gallic acid (792.9 kJ/mol [46]) and caffeic acid (762.9 kJ/mol) calculated at the same level of theory are **La**_**1**_, **La**_**2**_, and **La**_**6**_. Furthermore, the IPs for **La**_**1-3**_ and **La**_**6**_ are lower than that of ascorbic acid in gas phase and in water (804. 000 kJ/mol (gas phase) and 517.484 (water) kJ/mol at B3LYP/6-311++G(2d,2p)//B3LYP/6-31G(d,p) [41]. On the whole, this denotes the higher antioxidant power of molecules (**La**_**1-3**_ and **La**_**6**_**)** compared to that of ascorbic acid in these two media.

In solution phase, IP values are lower than the matching gas phase values. The average differences between IP in gas and that in other media are equal to 267.611 kJ/mol (water), 253.931 kJ/mol (acetonitrile), 90.930 kJ/mol (benzene), and 97.808 kJ/mol (toluene). This demonstrates the sensitivity of the cation radical forms of isolated ligand (**La**_**i**_) to the polarity of different solvents used [21, 47]. This may be attributed to the lowest value of the enthalpies of solvation of the electron. The increasing order of IP in media is water < acetonitrile < toluene < benzene < gas. From [Supplementary-material supplementary-material-1], the IPFE values calculated are indicating that SET mechanism is not spontaneous regardless of the environment considered. The computed IP_C_ values of Fe^2+^ chelates in three media (gas, water, and benzene) are reported according to Koopman's theorem (as above indicated) in [Supplementary-material supplementary-material-1]. The comparison of this IPC values of complexes to those of free ligands (Tables [Supplementary-material supplementary-material-1] and [Supplementary-material supplementary-material-1]) displays the facts that the values of the former are greatly higher. The difference in IP is in the following range: 107.934–712.718 (gas phase), 36.810–632.220 (acetonitrile), and 280.561–407.583 kJ/mol (benzene).

This is an indication that the electron extraction from **Fe**^**2+**^**...La**_**i**_ complexes is more difficult than in that from free ligand. This close-fitting binding of electron in cationic **Fe**^**2+**^complex system is similar to the computational prediction done for Fe^2+^-caffeic acid phenethyl ester derivative complexes (in gas phase) [48] and Fe^2+^-curcumin complexes (in gas and DMSO solvent phase) [24]. In the latter case, other divalent metal cations (Mn^2+^ and Zn^2+^) have shown their ability to enhance the antioxidant activity of isolated curcumin.

## 4. Conclusion

In the present paper, we have presented the computational calculations at B3LYP/6-311++G(d,p) level of theory of the lapachol and seven of its derivatives. The integral equation formalism of polarizable continuum model **(**IEF-PCM**)** was used to examine for predictions in solution phase (water, acetonitrile, benzene, and toluene). We limited our examination on SET mechanism. It turns out that this mechanism is not spontaneous regardless of the environment considered. The increasing order of IP yielded in media is ordered as water < acetonitrile < toluene < benzene < gas. IPs of Fe^2+^ complexes are greater than those of free isolated ligands. This fact exhibits the difficult electron extraction from **Fe**^**2+**^**...La**_**i**_ complexes. In general, the electron transfer from ligand to Fe^2+^ cation is more pronounced in tridentate mode coordination. In isolated ligands, the hydrogen bonding interactions have shown to be noncovalent in all the media, whereas the metal-ligand bonding is intermediate type interaction in gas phase. On the whole, the complexation process has shown to be highly exothermic. The optimized structures of **Fe**^**2+**^**...La**_**i**_ complexes at B3LYP/6-311++G(d,p) are characterized by Fe-O_i_ (*i* = 1, 2) bond lengths that are longer than Fe-C homologues in all the media.

## Figures and Tables

**Table 1:** Values (kJ/mol) of metal ion affinity (MIA), metal binding enthalpy, and metal binding free energy in various media at B3LYP/6-311++G(d,p).

	Gas			Acetonitrile			Benzene		
*Complexes*	MIA	Δ*H*_298_^0^	Δ*G*_298_^0^	MIA	Δ*H*_298_^0^	Δ*G*_298_^0^	MIA	Δ*H*_298_^0^	Δ*G*_298_^0^
**aLa**_**1**_	—	—	—	—	—	—	−448.997	−451.478	−411.684
**aLa**_**2**_	−1876.9	−1879.4	−1841.2	−135.203	−137.681	−97.466	—	—	—
**aLa**_**3**_	−976.6	−979.1	−935.9	—	—	—	—	—	—
**aLa**_**4**_	−956.6	−959.1	−922.2		−164.834		−412.429	−414.910	−369.907
**aLa**_**5**_	−961.8	−964.2	−928.2	−162.356	−1741.940	−116.344	−524.769	−527.248	−479.892
**aLa**_**6**_	−2558.1	−2560.6	−2524.5	−1739.462	−1739.462	−1701.550	−2038.123	−2040.602	−1998.901
**aLa**_**7**_	−2594.9	−1879.4	−2561.2	—	—	—	—	—	—
**aLa**_**52**_									
**aLa**_**53**_	1178.75	1181.2	−1131.1	−207.6	−210.100	−159.3			
**bLa**_**1**_	−2018.37	−201840.2	−201797.9	−146.868	−149.346	−99.155	—	—	—
**bLa**_**2**_	−941.0	−943.5	−1841.2	−51.541	−540.017	−8.210	—	—	—
**bLa**_**3**_	−1105.1	−989.7	−2254.5	—	—	—	—	—	—
**bLa**_**6**_	−2612.5	2208.5	−2574.6	—	—	—	—	—	—
**bLa**_**7**_	−10595.28	−1153.7	−105911.0	—	—	—	—	—	—
**cLa**_**1**_	−1152.3	−115478.7	−1106.8	−190.365	−192.843	−141.272	−564.464	−566.945	−512.030
**cLa**_**2**_	−1101.3	−1103.7	−1058.3	−169.374	−171.852	−126.575	−521.939		−443.670
**cLa**_**3**_	−1118.3	1120.8	−1073.1	—	—	—	—	—	—
**cLa**_**6**_	−1198.5	−1201.0	−1161.7	—	—	—	−618.392	−620.870	−577.61
**cLa**_**7**_	−105980.9	−105983.4	−105938.6	−104253.8	−104256.3	−104210.4	−105363.7	−105366.1	−105316.6

**Table 2:** Values of ligand...Fe^2+^ distance, NBO charge carried by Fe^2+^ cation, and topological parameters of ligand...Fe^2+^ interactions at B3LYP/6-311++G(d,p) (bold face numbers related to X…Fe^2+^ interaction and lower italicized numbers related to Y⋯Fe^2+^ interaction).

Complexes	**Ligand...Fe** ^**2+**^ **separation** distance (Å)	Q/e	*E*_int_	*ρ*(*r*) (au)	∇^2^*ρ*(*r*) (au)	*H*(*r*) (kJ/mol)	*G*(*r*) (kJ/mol)	*V*(*r*) (kJ/mol)	*λ*_1_	*λ*_2_	*λ*_3_	*ɛ*
**k** ^**2**^ **-O**_**1**_ **,O**_**2**_												
**aLa**_**2**_	1.908 1.981	1.432 **1.422**	−1876.9 –	0.0880 0.0690	0.618 0.486	−6.120 5.970	411.805 313.125	−417.924 −307.151	−0.0447 0.6175	0.7587 −0.0458	−0.0959 −0.0855	1.146224 0.866129
**aLa**_**3**_	1.843 1.921	1.008	−976.6	0.1090 0.0860	0.720 0.564	−36.700 −10.430	509.125 380.935	−545.829 −391.364	0.9478 −0.0670	−0.0820 0.7692	−0.1460 −0.1377	0.780231 1.054578
**aLa**_**4**_	1.835 1.922	1.027 *1.234*	−956.6	0.1110 0.0850	0.736 0.561	−41.019 −9.506	524.223 377.715	−565.243 −387.221	−0.0802 0.7740	0.9712 −0.0712	−0.1548 −0.1418	0.930247 0.991469
**aLa**_**5**_	1.844 1.937	0.990 –	−961.8 –	0.1070 0.8010	0.724 0.544	−33.340 −3.618	508.850 360.761	−542.190 −364.380	−0.0731 0.7237	0.9355 0.0724	−0.1380 −0.1175	0.887768 0.890006
**aLa**_**6**_	1.835 1.922	0.990 **1.421**	−2558.1 –	0.1110 0.0840	0.729 0.573	−39.050 −6.510	517.854 382.941	−556.905 −389.454	0.9616 −0.0658	−0.0791 0.7581	−0.1529 −0.1188	0.932448 0.804333
**aLa**_**7**_	1.851 1.947	0.963 –	−2594.9 –	0.1050 0.7700	0.715 0.536	−28.530 −0.129	497.552 351.920	−526.082 −352.048	−0.0686 0.6933	0.9111 −0.5591	−0.1280 −0.1015	0.866595 0.815607
**k** ^**2**^ ** −O**_**2**_ **,O**												
**aLa**_**52**_	1.786 1.884	0.767 **1.046**	−1151.2 −	0.2870 0.3300	−0.801 −0.945	−864.007 −921.327	337.436 300.811	−1200.786 −1222.138	0.3667 0.3617	−0.5037 −0.7243	−0.6643 −0.5828	0.318769 0.242798
**k** ^**2**^ ** −O**_**3**_ **,O**												
**aLa**_**53**_	1.808 1.871	0.839 **1.130**	−1178.7 −	0.1190 0.0980	0.802 0.662	−53.830 −19.278	580.507 454.053	−634.338 −473.331	1.0499 **−0.1565**	−0.096 **−0.5406**	−0.1518 0.8729	0.585220 1.893919
**k** ^**3**^ ** −(O**_**2**_ **,C=C)**												
**bLa**_**1**_	1.784 **2.004** *2.038*	0.618 **0.997** –	−20183.7 – –	0.1320 **0.1070** *0.0960*	0.764 **0.245** *0.2576*	−86.608 **−93.192** *−73.442*	588.395 **253.906** *242.528*	−675.003 **−347.099** *−315.969*	1.1899 **−0.1017** *−0.1015*	−0.2043 **0.4728** *0.4260*	−0.2212 **−0.1263** *−0.0669*	0.082728 **0.241748** *0.516271*
**bLa**_**2**_	2.053 **1.980**	0.674 **1.002** *0.820*	−941.0 – –	0.0527 **0.1139** –	0.429 **0.3026** –	−17.738 **−10.454** –	263.930 **303.147** –	−246.192 **−407.689** –	0.4645 **−0.1129** –	−0.0233 **0.5230** –	−0.0121 **−0.1075** –	0.919931 **0.049950** –
**bLa**_**3**_	1.772 **2.039** *1.981*	0.538 – –	−1105.1 – –	0.1389 **0.1022** *0.1179*	0.7834 0.2961 0.2555	−100.780 −80.968 −115.288	614.954 275.341 282.988	−715.734 −356.309 −398.276	1.2696 −0.0569 −0.1461	−0.2022 0.4603 0.5267	−0.2840 −0.1072 −0.1251	0.404762 0.883927 0.168423
**bLa**_**6**_	1.918 1.919 2.325	0.607 – –	−2612.5 –	0.0885 0.1343	0.5752 0.2583	−11.925 −148.704	389.493 318.259	−401.418 −466.963	0.7561 −0.1922	−0.1154 0.6103	−0.6549 −0.1598	0.762316 0.202276
**bLa**_**7**_	1.808 2.119 2.025	0.521 **0.843** –	−105952.8 − −	0.1264 0.8184 0.1015	0.6941 0.2114 0.1803	−77.219 −52.574 −87.858	532.792 191.359 206.175	−610.011 −243.933 −294.033	1.1400 −0.6246 −0.1288	−0.1991 0.3506 0.4422	−0.2468 −0.0767 −0.1331	0.239906 0.228563 0.032791
**k** ^**2**^ ** −(O**_**3**_ **,C=C)**												
**cLa**_**1**_	1.799 2.003 2.040	0.618 **0.967** –	−1152.30 – –	0.1240 0.1078 0.0965	0.7783 0.2088 0.2382	−69.056 −95.037 −74.481	579.899 232.059 230.842	−648.955 −327.097 −305.323	1.1161 −0.1074 −0.1079	−0.2081 0.4731 0.4266	−0.1297 −0.1570 −0.0805	0.603944 0.462709 0.341246
**cLa**_**2**_	1.788 2.156 2.119	1.016 **1.046** *0.848*	−1101.3 – –	0.1303 0.0759 0.0840	0.8045 0.1458 0.1610	−81.455 −48.375 −61.404	609.483 144.063 167.050	−690.936 −192.437 −228.454	0.1162 −0.0663 −0.1038	−0.1942 0.3069 0.3440	−0.1638 −0.9477 −0.0792	0.185365 0.428508 0.311229
**cLa**_**3**_	1.794 2.037 1.984	0.513 – –	−1118.3 – –	0.1267 0.1012 0.1175	0.782 0.286 0.245	−74.266 −79.636 −114.417	587.632 267.377 275.450	−661.899 −347.013 −389.866	1.1585 −0.0544 −0.1450	−0.1937 0.4481 0.5182	−0.1827 −0.1076 −0.1269	0.060095 0.976210 0.150727
**cLa**_**6**_	1.865 2.080 2.098	0.948 – –	−1198.5 – –	0.1046 0.0910 0.0858	0.6198 0.2369 0.2252	−35.057 −65.729 −58.587	441.884 221.198 206.406	−476.941 −286.927 −264.993	0.9067 −0.0935 −0.1007	−0.1091 0.3927 0.3712	−0.1778 −0.6229 −0.0453	0.630515 0.501621 1.223375
**cLa**_**7**_	1.821 2.083 2.014	0.521 **0.521** *1.062*	−105980.9 – –	0.1171 0.0491 0.0887	0.6875 0.1972 0.2172	−57.602 −17.174 −62.854	508.853 146.584 205.422	−566.455 −163.758 −268.275	1.0486 −0.0464 −0.7478	−0.2032 −0.0677 0.3783	−0.1579 0.3113 −0.8634	0.287284 0.458724 0.154464

## Data Availability

The data used to support the findings of this study are available.

## Supplementary Materials

Supplementary Materials Table 1S: intermolecular hydrogen bond parameters; BCP parameters: electron density ( ) and Laplacian of the electron density ( ) and hydrogen bond strength (kJ/mol) for lapachol and its derivatives at B3LYP/6-311++G(d,p)//B3LYP/6-31+G(d,p) level in other media. Table 2S: values in kJ/mol of ionization potential (bold face numbers are calculated using Koopman theorem) and ionization potential free energy of free ligands in various media calculated at B3LYP/6-311++G(d,p). Table 3S: values in kJ/mol of ionization potential and ionization potential free energy of Fe2± ligand complexes in various media calculated at B3LYP/6- 311++G(d,p)//B3LYP/6-31+G(d,p). Figure 1S: linear dependence of H-bond distances at bond critical points in the gas phase with electron density and Laplacian of electron density.
