# Supplementary material for: Antioxidant Properties of Lapachol and Its Derivatives and Their Ability to Chelate Iron (II) Cation: DFT and QTAIM Studies
Source: Bioinorg Chem Appl. 2020 Mar 31;2020:2103239. doi: 10.1155/2020/2103239 (PMC7150675; doi:10.1155/2020/2103239)
Supplement: Supplementary Materials — Table 1S: intermolecular hydrogen bond parameters; BCP parameters: electron density ( ) and Laplacian of the electron density ( ) and hydrogen bond strength (kJ/mol) for lapachol and its derivatives at B3LYP/6-311++G(d,p)//B3LYP/6-31+G(d,p) level in other media. Table 2S: values in kJ/mol of ionization potential (bold face numbers are calculated using Koopman theorem) and ionization potential free energy of free ligands in various media calculated at B3LYP/6-311++G(d,p). Table 3S: values in kJ/mol of ionization potential and ionization potential free energy of Fe2± ligand complexes in various media calculated at B3LYP/6- 311++G(d,p)//B3LYP/6-31+G(d,p). Figure 1S: linear dependence of H-bond distances at bond critical points in the gas phase with electron density and Laplacian of electron density. [file 2103239.f1.pdf]

**Table 1S**: Intermolecular hydrogen bond parameters, BCP parameters: electron density ($\rho(r)$) and Laplacian of the electron density ($\nabla^2\rho(r)$) and hydrogen bond strength (kJ/mol) for lapachol and its derivatives at B3LYP/6-311++G(d,p)//B3LYP/6-31+G(d,p) level in other media.

**Table 2S**: Values in kJ/mol of ionization potential (bold face numbers are calculated using Koopman theorem) and ionization potential free energy of free ligands in various media calculated at B3LYP/6-311++G(d,p).

**Table 3S**: Values in kJ/mol of ionization potential and ionization potential free energy of $Fe^{2+}$- ligand complexes in various media calculated at B3LYP/6-311++G(d,p)//B3LYP/6-31+G(d,p)

**Figure 1S**: Linear dependence of H-bond distances at bond critical points in the gas phase with electron density and Laplacian of electron density.

**Table 1S**: Intermolecular hydrogen bond parameters, BCP parameters: electron density ($\rho(r)$) and Laplacian of the electron density ($\nabla^2\rho(r)$) and hydrogen bond strength (kJ/mol) for lapachol and its derivatives at B3LYP/6-311++G(d,p) level in other media.

| **Species** | **H-bond parameters** | | | **BCP parameters** | | | | | |
|---|---|---|---|---|---|---|---|---|---|
| | $O_2$-$H_1$...$O_1$ (Å) | $O_2$...$O_1$ (Å) | $O_2$-$H_1$...$O_1$ Angle(°) | $\rho(r)$ (au) | $\nabla^2\rho(r)$ (au) | $H_{BCP}(r)$ (kJ/mol) | $G_{BCP}(r)$ (kJ/mol) | $V_{BCP}$ (kJ/mol) | $E_{int}$ (kJ/mol) |
| **Vacuo** | | | | | | | | | |
| **La1** | 2.009 | 2.609 | 117.6 | 0 .023 | 0.099 | 7.50 | 57.38 | -49.88 | 24.94 |
| **La2** | 1.966 | 2.583 | 118.8 | 0.025 | 0.107 | 7.20 | 62.73 | -55.53 | 27.76 |
| **La3** | 2.013 | 2.609 | 117.2 | 0.023 | 0.099 | 7.56 | 57.11 | -49.55 | 24.78 |
| **La4** | 2.011 | 2.610 | 117.5 | 0,023 | 0.099 | 7.50 | 57.26 | -49.76 | 24.88 |
| **La5** | 2.012 | 2.610 | 117.5 | 0.023 | 0.099 | 7.50 | 57.20 | -49.70 | 24.85 |
| **La6** | 1.966 | 2.584 | 118.8 | 0.026 | 0.112 | 9.08 | 64.41 | -55.33 | 27.66 |
| **La7** | 1.928 | 2.561 | 120.0 | 0.028 | 0.117 | 8.27 | 68.57 | -60.30 | 30.15 |
| **La8** | 2.012 | 2.378 | 117.4 | 0.023 | 0.098 | 7.20 | 57.21 | -49.69 | 24.84 |
| **Water** | | | | | | | | | |
| **La1** | 2.027 | 2.615 | 116.7 | 0.023 | 0.094 | 4.98 | 56.53 | -51.56 | 25.78 |
| **La2** | 1.986 | 2.590 | 117.8 | 0.025 | 0.110 | 9.43 | 62.46 | -53.04 | 26.52 |
| **La3** | 2.030 | 2.615 | 116.4 | 0.022 | 0.096 | 7.64 | 55.48 | -47.84 | 23.92 |
| **La4** | 2.018 | 2.612 | 117.1 | 0.023 | 0.107 | 10.11 | 59.96 | -49.86 | 24.93 |
| **La5** | 2.027 | 2.615 | 116.6 | 0.023 | 0.106 | 10.34 | 59.55 | -49.22 | 24.61 |
| **La6** | 1.985 | 2.590 | 117.8 | 0.025 | 0.110 | 9.40 | 62.52 | -53.12 | -26.56 |
| **La7** | - | - | - | - | - | - | - | - | - |
| **Acetonitrile** | | | | | | | | | |

| | | | | | | | | | |
|---|---|---|---|---|---|---|---|---|---|
| **La1** | 2.026 | 2.615 | 116.7 | 0.023 | 0.107 | 10.33 | 59.63 | -49.30 | 24.65 |
| **La2** | 1.985 | 2.590 | 117.8 | 0.025 | 0.110 | 9.42 | 62.50 | -53.08 | 26.54 |
| **La3** | 2.030 | 2.615 | 116.4 | 0.022 | 0.096 | 7.64 | 55.50 | -47.86 | 23.93 |
| **La4** | 2.026 | 2.615 | 116.7 | 0.023 | 0.107 | 10.30 | 59.62 | -49.32 | 24.66 |
| **La5** | 2.027 | 2.615 | 116.7 | 0.023 | 0.106 | 10.33 | 59.56 | -49,24 | 24.62 |
| **La6** | 1.985 | 2.590 | 117.8 | 0.025 | 0.109 | 9.40 | 62.56 | -53.16 | 26.58 |
| **La7** | 1.938 | 2.564 | 119.3 | 0.027 | 0.115 | 8.45 | 67.34 | -58.90 | 29.45 |
| **La8** | 2.027 | 2.615 | 116.6 | 0.023 | 0.106 | 10.35 | 59.52 | -49.18 | 24.59 |
| **Benzene** | | | | | | | | | |
| **La1** | 2.018 | 2.612 | 117.2 | 0.023 | 0.107 | 10.12 | 59.99 | -49.88 | 24.94 |
| **La2** | 1.974 | 2.586 | 118.4 | 0.025 | 0.111 | 9.20 | 63.60 | -54.40 | 27.20 |
| **La3** | 2.020 | 2.611 | 116.9 | 0.023 | 0.107 | 10.20 | 59.86 | -49.66 | 24.83 |
| **La4** | 2.018 | 2.612 | 117.2 | 0.023 | 0.107 | 10.10 | 59.97 | -49.87 | 24.94 |
| **La5** | 2.019 | 2.612 | 117.1 | 0.023 | 0.107 | 10.11 | 59.90 | -49.79 | 24.90 |
| **La6** | 1.974 | 2.586 | 118.3 | 0.025 | 0.111 | 9.22 | 63.55 | -54.33 | 27.17 |
| **La7** | 1.933 | 2.562 | 119.7 | 0.027 | 0.116 | 8.36 | 67.98 | -59.62 | 29.81 |
| **La8** | 2.019 | 2.611 | 117.0 | 0.023 | 0.107 | 10.11 | 59.89 | -49.78 | 24.89 |
| **Toluene** | | | | | | | | | |
| **La1** | 2.018 | 2.612 | 117.1 | 0.023 | 0.107 | 10.13 | 59.97 | -49.86 | 24.93 |
| **La2** | 1.975 | 2.587 | 118.3 | 0.025 | 0.111 | 9.24 | 63.51 | -54.26 | 27.13 |
| **La3** | 2.021 | 2.611 | 116.9 | 0.023 | 0.107 | 10.20 | 59.84 | 59.84 | 29.92 |
| **La4** | 2.018 | 2.612 | 117.1 | 0.023 | 0.107 | 10.11 | 59.96 | -49.84 | 24.92 |
| **La5** | 2.020 | 2.613 | 117.1 | 0.023 | 0.107 | 10.14 | 59.88 | -49.74 | 24.87 |
| **La6** | 1.985 | 2.590 | 117.8 | 0.025 | 0.110 | 9.41 | 62.52 | -53.10 | 26.55 |
| **La7** | - | - | - | - | - | - | - | - | - |
| **La8** | 2.019 | 2.611 | 117.0 | 0.023 | 0.107 | 10.12 | 59.87 | -49.76 | 24.88 |

**Table 2S**: Values in kJ/mol of ionization potential (bold face numbers are calculated using Koopman theorem) and ionization potential free energy of free ligands in various media calculated at B3LYP/6-311++G(d,p)

| IP | gas | water | acetonitrile | Benzene | toluene |
|---|---|---|---|---|---|
| **La$_1$** | 764.246 | 497.560 | 510.086 | 665.984 | 656.838 |
| | **627.022** | **638.527** | **638.049** | **630.488** | **630.698** |
| **La$_2$** | 754.406 | 486.339 | 498.848 | 657.115 | 647.613 |
| | **616.231** | **622.979** | **622.716** | **615.233** | **618.279** |
| **La$_3$** | 800.905 | 507.370 | 519.720 | 704.240 | - |
| | **667.428** | **679.663** | **679.348** | **672.522** | **672.758** |
| **La$_4$** | 810.945 | - | 554.851 | 711.985 | 702.877 |
| | **676.040** | **676.119** | **676.959** | **676.092** | **676.119** |
| **La$_5$** | 806.639 | 551.425 | - | 710.444 | 701.501 |
| | **683.418** | **684.423** | **684.179** | **683.102** | **683.288** |
| **La$_6$** | 749.630 | 480.011 | 492.645 | - | 653.205 |
| | **691.268** | **693.158** | **693.027** | **691.373** | **691.425** |
| **La$_7$** | 751.255 | **-676.854** | 500.148 | 652.993 | - |
| | **671.839** | | **677.038** | **673.336** | **673.467** |
| **La$_8$** | 823.044 | 553.213 | 566.009 | 724.782 | 715.781 |
| | **691.084** | **690.900** | **690.139** | **690.297** | **690.244** |
| **IPFE** | **gas** | **water** | **acetonitrile** | **Benzene** | **Toluene** |
| **La$_1$** | 755.400 | 443.600 | 469.000 | 603.200 | 650.000 |
| **La$_2$** | 743.300 | 436.800 | 462.500 | 605.900 | 639.500 |
| **La$_3$** | 796.200 | 459.300 | 485.200 | 644.400 | |
| **La$_4$** | 800.9 | | 513.300 | 647.800 | 696.300 |
| **La$_5$** | - | 494.200 | - | 648.000 | 697.000 |
| **La$_6$** | 651.400 | 339.800 | 364.700 | 499.600 | 548.000 |
| **La$_7$** | 632.3 | 331.700 | 363.300 | 481.900 | - |
| **La$_8$** | - | 506.200 | 532.600 | 659.400 | 707.900 |

**Table 3S**: Values in kJ/mol of ionization potential and ionization potential free energy of $Fe^{2+}$- ligand complexes in various media calculated at B3LYP/6-311++G(d,p)

| $IP_C$ | Gas | Acetonitrile | Benzene |
|---|---|---|---|
| **$aLa_1$** | - | - | 911.259 |
| **$aLa_2$** | 724.165 | 733.197 | - |
| **$aLa_3$** | 1294.555 | - | - |
| **$aLa_4$** | 1355.939 | - | 1042.612 |
| **$aLa_5$** | 1285.970 | 753.466 | 1003.912 |
| **$aLa_6$** | 1355.834 | 743.384 | 1003.387 |
| **$aLa_7$** | 1192.948 | | |
| **$aLa_{52}$** | 1357.226 | - | - |
| **$aLa_{53}$** | 1348.404 | 768.878 | 1014.388 |
| **$bLa_1$** | 1340.344 | 721.566 | - |
| **$bLa_2$** | 1388.758 | 730.230 | - |
| **$bLa_3$** | 1368.489 | - | |
| **$bLa_4$** | 1388.758 | - | 1029.012 |
| **$bLa_5$** | 1357.226 | 720.989 | 1001.681 |
| **$bLa_6$** | 1394.587 | - | - |
| **$bLa_7$** | 1323.593 | - | - |
| **$cLa_1$** | 1337.508 | 757.772 | 1000.495 |
| **$cLa_2$** | 1368.935 | 775.336 | 1022.816 |
| **$cLa_3$** | 1346.041 | - | - |
| **$cLa_4$** | 1348.404 | - | - |
| **$cLa_6$** | 1363.422 | - | 1016.699 |
| **$cLa_7$** | 1309.258 | 1309.258 | 992.728 |

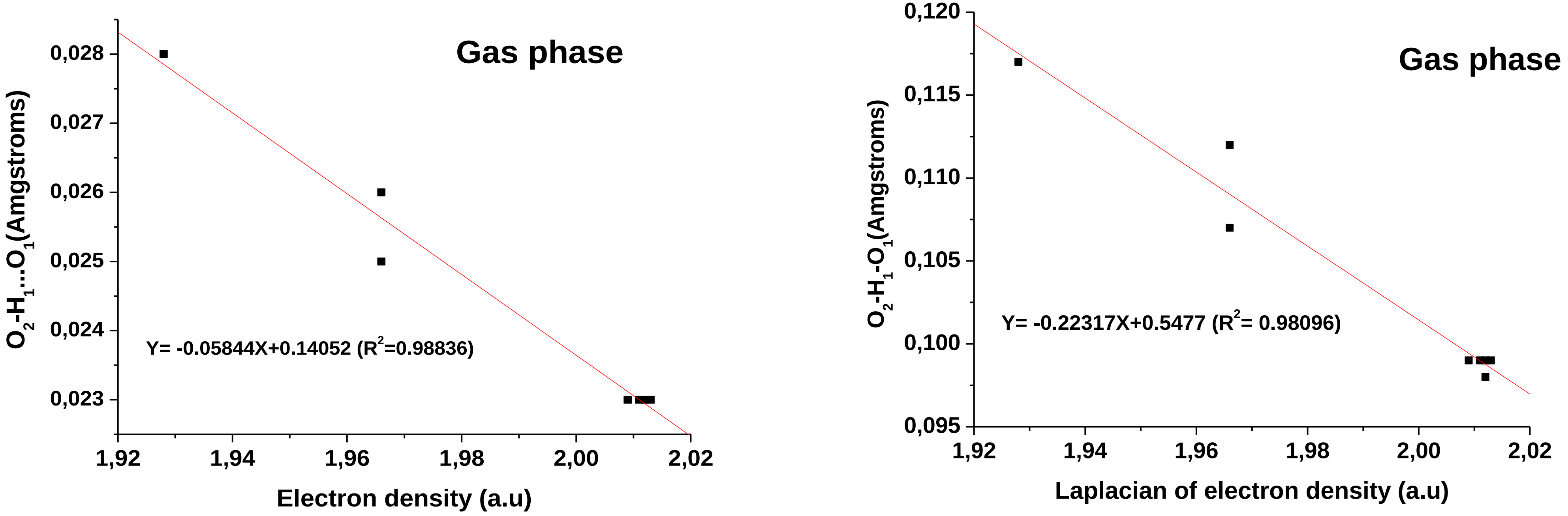


**Figure 1S**: Linear dependence of H-bond distances at bond critical points in the gas phase with electron density and Laplacian of electron density.

# SUPPLEMENTARY MATERIALS DESCRIPTION

From the B3LYP/6-311++G(d,p) of isolated ligands, the topological parameters of Intermolecular hydrogen bond formed (Table 1S) have been calculated using the quantum mechanics atom in molecule (QMAIM) approach. Independently of the media, the formation of hydrogen bond ($O_2$-$H_1$…$O_1$ bond) obeys to to the cut-of definition of X-H…$O_2$ hydrogen bond (H…O2 < 3.0Å and X-H… O2 angles > 110° [1]). The positive values values of Laplacian of electron density ($\nabla^2 \rho(r)$) is an indication that the hydrogen bonding interaction is dominated by the local excess in the kinetic energy. The non-covalent nature of this bonding interaction is confirmed by the average ratio $-G_{BCP}(r)/V_{BCP}(r)$ greater than 1 in various media: (gas (1.15), water (1.14), acetonitrile (1.18), benzene (1.19) and toluene (1.16)). HB strength obtained that range from 23,92 to 30.15 kJ/mol are following the range of normal HBs (8,36-41,8 kJ/mol) [2]. Fig 1S reveals the linear correlation between the bond distance and the two energetic parameters (electron density and Laplacian of electron density). The calculated IP values obtained directly from Koopman's theorem for isolated ligand examined are lower in gas phase. In solution phase, IP values are lower than the matching gas phase values.

## References


[1] T. Steiner, "Te Hydrogen Bond in the Solid State," *Angewandte Chemie International Edition*, vol. 41, no. 1, pp. 48‑76, 2002.

[2] Tz.Mihaylov, N.Trendafilova, I.Kostova, I.Georgieva, G.Bauer, "DFT modeling and spectroscopic study of metal-ligand bonding in La(III) complex of coumarin-3-carboxylic acid",*Chemical Physics,* vol 327, pp209-219, 2006.
